# Assessing Order in Liquid, Supercooled, and Crystalline Water

**DOI:** 10.1021/acs.jpcb.5c08791

**Published:** 2026-05-02

**Authors:** Rajendra Maharjan, Casey Williamson, Christopher J. Fennell

**Affiliations:** † Department of Chemistry, 7618 Oklahoma State University, Stillwater, Oklahoma 74078, United States; ‡ Department of Chemistry, 3644 Grinnell College, Grinnell, Iowa 50112, United States

## Abstract

Water has strong directional interactions and forms a network of hydrogen bonds (H-bonds) in aqueous solutions. In order to assess the nature of this network as a function of the thermodynamic state, a generalized approach is introduced for enumerating the extended connectivity of H-bonding paths. By identifying and classifying rings formed from H-bonds where the donor-to-acceptor directionality can be considered, a ring summation factor metric can be constructed in a form comparable to the commonly used tetrahedral order parameter. This factor ranges between 0, where there is no closed path ring connectivity, and 1, where all potential H-bonds participate in unique closed paths. This new metric is used to explore the relative limits of water ordering in liquid, supercooled, and crystalline condensed phase systems. In liquid systems, network ordering is shown to depend on the specific state parameters and how different models for water perform at and outside of their commonly used conditions. As they are supercooled, water models that converge on a similar preferred crystalline form show a common ring distribution signature, indicating a universal characteristic ordering in the liquid environment preceding nucleation and crystal growth processes. The various crystalline ice polymorphs are observed to have unique degrees of order and distributions of ring sizes, and even different degrees of proton ordering can be identified using this approach. The detailed findings enable the identification of and subsequent remedy of potential deficiencies in tetrahedrality when characterizing the structure of crystalline water.

## Graphical abstract

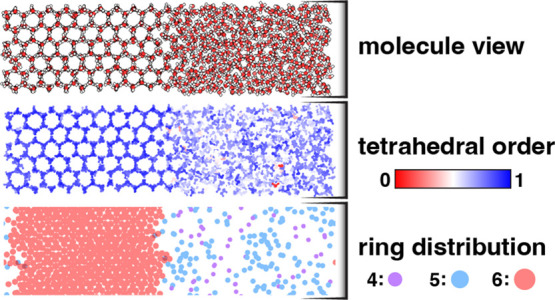

## Introduction

Water shows anomalous behavior that deviates from typical liquids in both thermodynamic and transport properties, the best known example being the temperature of maximum density in the liquid state.^1–9^ The origin of water’s unique behavior comes from water’s strong pairwise attractions through H-bonding, exhibiting complex molecular arrangements in the solid, liquid, or gaseous states.^8,10^ The H-bond network in aqueous solutions exhibits structural fluctuations, with individual water molecules adopting local arrangements of varying order over time due to molecular motions at a particular system’s temperature and pressure.^11,12^ The ideal local geometry about a water molecule is tetrahedral on average under ambient conditions due to the tetrahedral electron domain geometry about its central oxygen atom.^13–15^ However, asymmetry in water’s electron density makes nontetrahedral coordination geometries accessible, giving rise to chain-like and/or ring-like structures in extended arrangements of water molecules.^16–20^ Identifying and characterizing the local structure from such building blocks provides a critical link to the anomalous nature of water’s properties, and understanding this local structuring has motivated the development of various order parameters and metrics that reflect the structure of liquid water.^7,21–23^

Simplified and computationally efficient order parameters have been used to describe the structure of liquids, the transition between liquid and crystalline phases, anomalous properties of liquid water, and changes in liquid structure in the environment around dissolved solutes.^24–31^ Many types of order parameters have been developed including distance-based translational order parameters,^32^ the local structure index, LSI,^11^ to determine inhomogeneity in the distribution of radial distances from a given water molecule, Voronoi tessellation methods for geometrical analyses of local molecular arrangements,^33,34^ and the “*d*_5_” parameter that is based on the distance to the fifth nearest neighboring water molecule.^35^ One of the most popular order parameters used in such investigations is the tetrahedral order parameter, first proposed by Chau and Hardwick,^32^ but later revised to its current form by Errington and Debenedetti.^21^ The tetrahedral order parameter provides a simple numerical estimate of the averaged local order around each water molecule by calculating all possible angles formed between four nearest-neighboring waters through a central target water molecule. A maximum value of 1 represents a geometrically ideal tetrahedral arrangement of the four potential H-bonds.^21^

In its crystalline form, a sample of water is expected to show a tetrahedrality close to this maximum value of 1, given the presence of four near-ideal hydrogen bonds per water molecule in hexagonal ice, ice Ih. At different pressure and temperature state-points, ice can restructure into various crystalline polymorphs. There are various stable and metastable ice polymorphs, from ice Ih to ice XXI, that have been discovered experimentally, along with numerous additional forms predicted computationally.^36–39^ These polymorphs span both proton ordered and disordered forms, with the disordered forms involving randomized water molecule orientations in the crystal lattice satisfying the ice rules.^13,40^ Observing transitions between proton ordered-disordered phases is challenging due to the cooperative reorientation dynamics of the water molecules and the insensitivity of typical order parameters to the ordered–disordered nature of the crystal form.^38,41–43^ Monitoring the evolution of H-bond ring paths that retain information on the directionality of these interactions could be a strategy for dynamic identification of these phases.

Several nonequivalent closed-path enumeration techniques have been developed to characterize the topology of the H-bond network in condensed phase systems. These techniques derive from King’s approach for identifying closed paths (rings) in amorphous silicon dioxide and are typically modified for use with H-bond networks rather than covalently bonded networks.^44–47^ Some pruning criteria are necessary to restrict the exponential growth of the number of rings with increasing ring size. The criteria for generating complete ring statistics depend on reproducibility and comparability with already existing structures or reference systems.^48,49^ This requires an exhaustive and unambiguous ring closure scheme that enumerates minimal-sized rings without repetition and identifies bridges forming intersecting rings.^46^ Existing ring enumeration techniques are useful for gaining insight into the structural changes underlying the kinetics and mechanism of ice nucleation, liquid polymorphism in the supercooled state, and polyamorphism in amorphous states.^4,7,8,50–70^ Early related efforts by Rahman and Stillinger involved enumerating nonshort-circuited H-bond rings in liquid water, wherein no two vertices are connected by an H-bond to form smaller rings within larger rings.^10^ Speedy et al. later defined primitive rings as those in which no adjacent pair of sides (formed by three vertices) is shared in a way that leads to shorter H-bond paths, with each water molecule precisely forming four H-bonds if H-bonded to more than four water molecules.^71^ The fundamental difference between nonshort-circuited and primitive ring enumeration is schematically shown in Figure 1. Out of these early works, different approaches have been suggested to extensively investigate ring statistics for molecular arrangements in liquid and supercooled water, as well as structural changes occurring during phase transitions in supercooled and amorphous states at low temperatures.^45,72–75^

**Figure 1:**
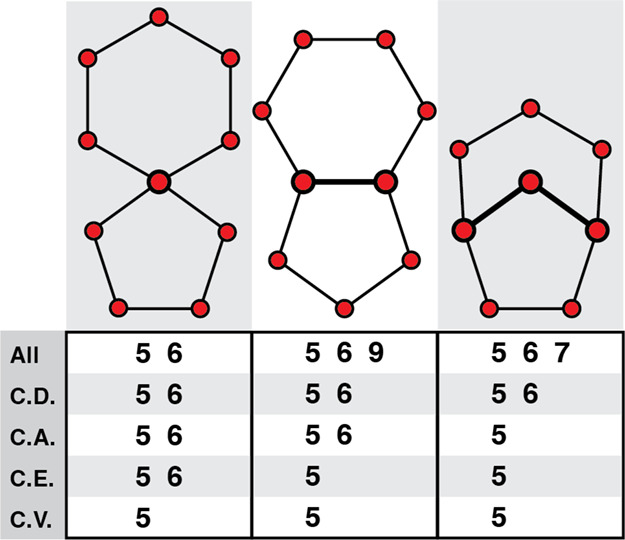
First row of the table, marked with “All” lists the sizes of all rings identified without pruning. Subsequent rows give the ring statistics after applying various pruning methods. The methods for pruning based on commonality are increasingly selective in identifying the shortest closed H-bonding paths. In all these 5- and 6-membered ring path cases, the common vertex (CV) method eliminates paths of length greater than five, since the rings in these examples all share at least one water molecule in common. The common dihedral (CD) method only eliminates perimeter rings, which share four sequential water molecules with the smaller interior rings. In contrast, all three structures have one 6- and one 5-membered nonshort-circuited ring according to Rahman and Stillinger’s definition; the middle and right structures, however, possess shorter connecting paths that prune 9- and 7-membered rings, respectively.

In this study, we discuss an algorithmic method for enumerating rings using different pruning criteria, as well as ways to identify directional rings. We introduce a new order parameter called ring summation factor (RSF), and compare it with a popular tetrahedral order parameter to assess local structural arrangements in liquid, supercooled, and crystalline ice. The computational method section presents the methods employed to build liquid, supercooled, and crystalline systems. In the results and discussion section, we discuss a way to enumerate directional rings and examine the unique ring distributions of different water models in the liquid and supercooled phases using different ring pruning criteria. Additionally, we study proton ordered-disordered ice polymorphs with respect to the directional rings and show ways to develop a distinctive fingerprint for every ice polymorph. Finally, we illustrate the implication of order parameters to study the kinetics and mechanism of water crystallization.

## Algorithmic Method for Enumerating Rings

Liquid water consists of a macroscopic H-bond network that frequently undergoes topological reformation due to its strained structure.^23^ In order to identify extended ordering in H-bonding systems, we developed an efficient algorithm that identifies continuous chains of H-bonded water molecules in aqueous systems and enumerates chains that return to the initial water, herein referred to as rings.

The first step in identifying rings is to build the H-bond network for the system of interest. The identification and population of rings depend upon the H-bond definition used for identifying H-bonds, with more relaxed definitions identifying rings that less tolerant definitions would not. These definitions have traditionally been based on some combination of geometry and energy criteria.^76,77^ Geometric definitions have the advantage of retaining information on the donor and acceptor identities, which can be useful for characterizing H-bonds based on proton ordering. In this work, we use the common Luzar and Chandler geometric H-bond definition, which has a donor–acceptor oxygen separation distance threshold of 3.5 Å and an *∠*OOH cone angle threshold of less than 30°.^78^

Following the construction of a complete map of the H-bonds in a system, chains of continuous H-bond sequences are constructed via exhaustive branch search starting from every water molecule. A given chain forms a ring or polygon only when the starting water molecule bonds back to itself. The process of enumerating all possible rings in periodic systems can be computationally cumbersome because the chains extending from each water will grow combinatorially.^10,71,79^

This exhaustive process enumerates all possible rings in the system, but we are principally interested in unique primitive rings.^71^ For example, many rings share the same sets of water molecules, just with different starting molecules as shown in Figure 1. To avoid overcounting due to this ring degeneracy and retain only primitive rings, we consider four ring pruning criteria, namely common vertex (CV), common edge (CE), common angle (CA), and common dihedral (CD). These pruning methods respectively identify neighboring rings that share 1, 2, 3, or 4 sequential H-bonded water molecules and retain only the smallest rings with shared water sequences. These methods extend the study by Speedy et al., which defined primitive polygons as the smallest rings sharing a common angle, formed by three common adjacent vertices with larger rings.^71^ The Speedy et al. method is equivalent to the CA approach described here.

When enumerating rings, each sequence of H-bonds in the chain can be read forward and reverse, i.e. clockwise and counterclockwise, and the starting point of the path could originate from any one of the members. A pentagonal ring, for example, generates ten instances of the same closed path in an unbiased enumeration. Only one ring from this count is retained in the primitive ring set. Additionally, in small systems under periodic boundary conditions, paths can connect across unit cell faces and artificially populate the set of rings. Such artificial paths are removed by ensuring that any H-bond that crosses a unit cell wall is matched by a returning H-bond in the path that crosses the same unit cell wall.^10,71,79^ For the sake of computational efficiency and because primitive rings rarely occur beyond heptagons, the maximum chain length was limited to ten H-bonds, resulting in a maximum ring size of 10 members.

Each H-bond consists of a hydrogen atom covalently bonded to one water molecule and pointing toward the oxygen of a second water. This establishes a directionality, where the donor is taken as the water contributing the covalently bonded hydrogen atom to the H-bond and the acceptor being the other water molecule. A directional ring requires an unbroken sequence of donor-to-acceptor waters (or vice versa) in the path, so no single water molecule contributes either two or zero covalently bonded hydrogen atoms to the ring. The ring pruning methods can additionally incorporate directionality, with DCV, DCE, DCA, and DCD referring to directional common vertex, directional common edge, directional common angle, and directional common dihedral pruning methods, respectively. Figure 2 shows how the DCA ring pruning method differs from the CA method in the case of a pentagon and a hexagon sharing an edge. Both rings can be directional rings as in the left illustration, but this is not the most probable case. There are many ways that one or both of the rings, the middle and right illustrations of Figure 2, can have a break in the donor-to-acceptor series. Considering directionality significantly reduces enumerated rings, and it can potentially provide additional insight into the ordering within a condensed phase aqueous system. For example, enumeration of directional rings is a useful way to distinguish the proton ordered ordered ice polymorphs, where water molecules orientationally align in the crystal lattice, from proton disordered ice polymorphs.

**Figure 2:**
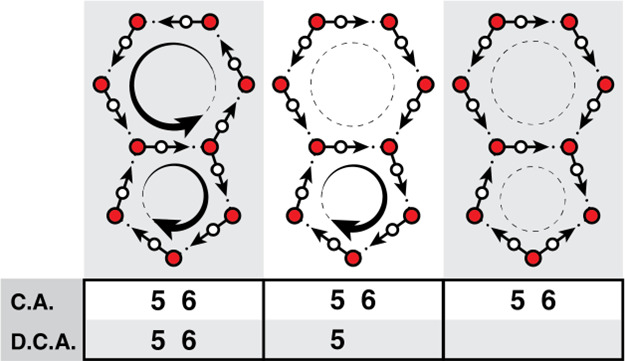
Directionality of the H-bonding paths can be further used to prune the H-bonding network. Neither of these 5- and 6-membered rings shares a common angle (CA), but depending on the sequence of donor (hydrogen atom) to acceptor (lone-pair) order, the paths are either retained or pruned.

To visualize the spatial distribution of rings, we generate and present POV-Ray images later in this article that place colored circles at the geometric center of each identified ring in ice crystals. The color and size of the circles represent the ring size, as specified in the corresponding figure captions. For clarity, these POV-Ray images show only slices of the crystals rather than their full depth. These pointillistic POV-Ray images highlight the unique densities and crystalline arrangements of water in ice, making them blueprints for identifying various ice polymorphs.

## Quantification of Order in Aqueous Systems

Originally proposed by Chau and Hardwick, and later revised by Errington and Debenedetti, the tetrahedral order parameter, *q*, is a tool for quantifying the local structure of water.^21,32^ The primary local interactions for each water molecule favor a tetrahedral arrangement in which it can ideally donate two and accept two H-bonds. The tetrahedral order parameter targets this local tetrahedrality for a given water molecule, *i*,

$$q=1-\frac{3}{8}\sum_{j=1}^{3}\sum_{k=j+1}^{4}{\left(\cos\;\Psi_{jk}+\frac{1}{3}\right)}^{2}$$ 1

where Ψ_*jk*_ is the angle between bond vectors *r*_*ij*_ and *r*_*ik*_, with *j* and *k* indicating the four nearest neighbor atoms of the same type. This form of *q* reports 0 for an ideal gas-like system and reaches a maximum of 1 for a regular tetrahedron, a value reported for a perfectly crystalline diamond lattice. Less ordered systems, like liquids and glasses, have values between 0 and 1. Changes in *q* with state variables have been widely used to investigate the structural properties of water.^5,21^ Recent advances in data analysis enable robust identification of structurally distinct local environments using appropriate order parameters or their combination.^54,80^

The average tetrahedrality, ⟨*q*⟩, can be computed to turn this initially local quantity into a global metric for determining order of a system. With a fully enumerated set of closed H-bond paths, we define an analogous metric for the entire system. By summing the counts of different sized primitive rings identified using the CA pruning criterion, a single-valued ring summation factor (RSF) quantifies the structural ordering of water in a system. RSF is defined as the total number of rings per maximum number of possible H-bonds,

$$\mathrm{RSF}=\frac{\mathrm{no}.\;\mathrm{of}\;\mathrm{rings}}{\mathrm{maximum}\;\mathrm{no}.\;\mathrm{of}\;\mathrm{H‐bonds}}$$ 2

All the identified primitive rings up to 10-membered rings are included in the “no. of rings”, as 10 is the maximum ring size identified in the network calculation. Each water molecule can donate two unique H-bonds to a crystalline water network, so the maximum number of possible H-bonds in any system is twice the number of water molecules. In crystalline water, like hexagonal ice, all these H-bonds participate in forming an equal number of hexagonal rings. Dividing by two times the number of water molecules in the simulation unit cell normalizes RSF to a limiting value of 1 when each water fully participates in a defect free crystal.

On the other extreme, a low density ideal gas-like system is characterized by a very low probability of forming rings due to the lack of successful H-bond paths, yielding a limiting RSF value of 0. In liquid or glassy states, the relative populations of rings depend on the specific pruning method employed. RSF requires a pruning technique to avoid redundancy and restrictive counting of only primitive rings, which would otherwise cause unbounded growth. The CA pruning method was employed to identify rings for all reported RSF values, and the ring sizes were limited to 10-membered rings in accordance with the network construction process. In this method, individual H-bonds rarely contribute to more than one ring, and all liquid and supercooled states yielded intermediate RSF values between 0 and 1. All pruning methods presented yield RSF values between 0 and 1 for both liquid and supercooled water.

While the average tetrahedral order parameter and RSF both have target ranges from 0 to 1, they quantify different aspects of water ordering. RSF involves enumeration of the CA pruned set of closed rings that include three or more water molecules. An RSF value therefore includes order from extended H-bonding connectivity and provides an overall picture of the network topology of the entire system. In contrast, *q* is derived from the immediate local structural ordering around a single water molecule, opening it up for use in enhanced sampling techniques that enforce local tetrahedrality. Numerically, RSF has a limiting low-order value of 0 as one cannot have fewer than zero closed H-bonding paths, while *q* can report negative values for highly distorted local geometries. Conversely, *q* has a limiting high order value of 1 due to the tendency of water toward forming local tetrahedral geometry on average as given by eq 1.

As RSF is a globally computed quantity for the system of interest, it depends on the construction and traversal of the complete H-bond network. We observe that the computational cost of RSF increases approximately quadratically with the number of H-bonds in the system, meaning RSF is slower to compute for lower temperature crystalline systems than for higher temperature liquid systems of similar size. Conversely, calculating the tetrahedral order parameter incurs minimal cost and typically scales linearly with system size once the particle neighborlist has been constructed. The associated code for RSF calculation also computes *q* with minimal increased computational burden.

## Computational Methods

Molecular dynamics (MD) simulation of liquid and supercooled water was performed using GROMACS/2021.4.^81^ Different water models, namely TIP3P, SPC, SPC/E, TIP4P, TIP4P-Ew, TIP4P/2005, TIP4P/Ice, and OPC, were used to study the liquid water configuration composed of 1000 water molecules.^82–88^ MD simulations were performed under isothermal–isobaric, *NpT*, conditions using periodic simple cubic simulation cells with varying temperatures and 1 atm pressure. These simulations were run for 200 ns, and the equations of motion were integrated every 2 fs time step using the leapfrog algorithm. The temperature coupling and pressure coupling were respectively controlled via a Nosé–Hoover thermostat using a time constant of 2 ps and via an isotropic Parrinello–Rahman barostat with a time constant of 10 ps.^89,90^ Molecular constraints were implemented with the LINCS algorithm at fourth order.^91^ The smooth particle mesh Ewald summation was adopted for considering the long-range electrostatic interactions with a PME grid spacing of 0.12 nm, a real-space cutoff of 1.0 nm, and an energy tolerance of 1 × 10^–5^ kJ/mol.^92^

For investigating different ice polymorphs, GenIce2/2.1.5 program was used to create the proton ordered and disordered crystals.^93,94^ The crystals were converted to GROMACS file formats, but they were otherwise used cleanly, not subjecting them to minimization or thermalization. Visual depictions of molecular structures and closed rings were generated using UCSF Chimera and POV-Ray/3.6.^95,96^

## Results and Discussion

### Occurrence of Directional Rings Approaches a Probabilistic Ideal with Increasing Ring Size

Directionality in H-bonding paths is inherently dependent upon proton ordering in aqueous systems, and identifying directional rings serves as a tool to distinguish collective proton ordering in an aqueous system. Directional rings occur naturally, so their presence alone does not necessarily guarantee the entire system is proton ordered. Instead, there exists an intrinsic probability of a directional ring from a random set of rings of the same size. The probability, *P*_D_, that a given ring is a directional ring is given by

$$P_{D}=2\times {\left(\frac{2}{3}\right)}^{n}$$ 3

where *n* is the number of H-bonds in the ring, 2/3 is the directional path continuation probability, and the number two is for the *C*_2_-symmetry of the ring directionality (clockwise or counterclockwise direction). The directional path continuation probability of 2/3 comes from the fact that each water molecule ideally forms 4 H-bonds. When a water molecule accepts an H-bond from the previous water in the chain, three sites remain (one acceptor and two donors). Subsequent water H-bonds to only the two donor sites of the three remaining H-bonding sites will continue this directional chain.

*P*_D_ is a predicted ideal probability assuming randomly connected H-bond ring paths. This randomness does not consider any steric or energetic effects influencing the chain connectivity that can occur in atomistic systems. An analog to this predicted probability can be directly observed from configurations of H-bonding systems using

$$P_{n,\mathrm{obs}}=\frac{\mathrm{no}.\;\mathrm{of}\;\mathrm{directional}\;\mathrm{rings}}{\mathrm{total}\;\mathrm{no}.\;\mathrm{of}\;\mathrm{rings}}$$ 4

where both directional rings and total number of rings of the same size are counted before pruning to avoid biased counting of rings. Pruning preferentially eliminates large nondirectional rings, artificially increasing the directional ring population for larger rings in particular.

Observed deviations of *P*_*n*,obs_ from *P*_D_ indicate the presence of a biasing force in ring formation. We calculated the observed fraction of directional rings, *P*_*n*,obs_, from liquid water simulations and compared it with the predicted *P*_D_ values. Figure 3 shows the comparison between predicted fraction of directional rings, *P*_D_, (orange curve) and observed fraction of directional rings, *P*_*n*,obs_, at 298.15 K liquid water simulation using TIP4P/Ice water model (blue curve) as a function of ring size. The largest deviation between the two curves is seen with the 3-membered rings, the triangles. Close to 60% of the triangles are predicted to be directional rings, yet only around 15% are observed as directional in simulated water. This discrepancy arises from enthalpic strain in forming a 3-member ring. Because ideal hydrogen bonding involves a nearly tetrahedral geometry around water molecules, interior angles of rings that deviate from 109.47° will require nonideal H-bonds. This strain is partly relieved when a water molecule in the ring either donates or accepts two H-bonds from other waters in the ring. The relief comes because the HOH angle used in rigid water models like OPC and TIP4P/Ice is less than 109.47°, a bit closer to the 60° interior angle of an equilateral triangle. 3- and 4-site water models also lack localized lone-pair sites, allowing them to more flexibly accept nonideal H-bonds.^97^ Double donation or acceptance of H-bonds ensures that the ring will not be a directional ring.

**Figure 3:**
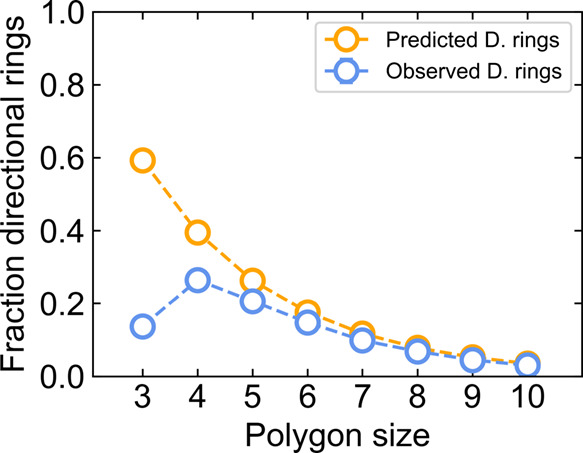
Predicted fraction of directional rings converges with the value measured in liquid water simulations for increasingly large ring shapes. The predicted probability of observing a directional ring from the total set of rings in a TIP4P/Ice simulation at 298.15 K is shown in orange, while the fraction of unpruned directional rings directly enumerated from the simulation is shown in blue.

A similar bias against directional rings is expected for 4-membered rings because the geometry at the vertices is not tetrahedral—the interior angles average 90°. The observed *P*_D_ decreases to 40% from the 59% in 3-membered rings because it is increasingly less likely to observe a continuous chain of H-bonds pointed in a common direction. However, *P*_*n*,obs_ in liquid state simulations increases from 14% for 3-membered rings to 26% for 4-membered rings. This is still less than the predicted *P*_D_ as nonideal, flexed/bent H-bonds are required to form a closed ring. As ring size increases, the predicted *P*_D_ continues to decrease to less than 5% for sizes of 10 or more. The *P*_*n*,obs_ also converges toward *P*_D_, as average angles between neighboring H-bonds in larger rings have greater freedom to approach tetrahedral geometries.

Removal of ring strain as a factor in the formation of closed paths will come primarily in the crystalline state. In solid forms, water molecules obey the Bernal–Fowler ice rules, and various ice phases will have fixed relative populations of ring sizes. Calculating the probability of directional rings in ice polymorphs thus reveals their peculiar structural features. Table 1 shows the probability of unpruned directional rings observed in ice Ih and ice III alongside the *P*_D_ and liquid water simulation results from Figure 3. While the observed fractions of all types of directional rings larger than 4-membered rings in liquid water slowly converge toward predicted *P*_D_ values because of reduced ring strain, only certain types of directional rings are characteristic of the ice polymorphs. The observed fractions of the rings in the indicated ice polymorphs overlap with the corresponding *P*_D_ values. Note that both ice Ih and ice III are proton disordered crystals.

**Table 1:** Entropically Predicted, *P*_D_, and Observed Fraction, *P*_*n*,obs_, of Directional Rings in TIP4P/Ice Simulations of Liquid Water at 298.15 K and Proton Disordered Ice Crystals from GenIce2, Where the Angle Bracketed Numbers Indicate Average Values for Rings of That Size and the Numbers in Parentheses Are the Measured Error in the Last Digit

phase	⟨3⟩	⟨4⟩	⟨5⟩	⟨6⟩	⟨7⟩	⟨8⟩	⟨9⟩	⟨10⟩
*P*_D_	0.593	0.395	0.263	0.176	0.117	0.078	0.052	0.035
liquid	0.14(2)	0.264(8)	0.206(4)	0.148(3)	0.098(2)	0.068(1)	0.045(1)	0.030(1)
ice Ih	0	0	0	0.176(6)	0	0.080(5)	0	0.036(2)
ice III	0	0	0.26(1)	0.18(3)	0.12(1)	0.077(3)	0.053(4)	0.035(3)

### Intermediate Ordering in Liquid and Supercooled Water Is Both Temperature- and Water Model-Dependent

Without ring pruning, there will be a dramatic growth in large rings due to the increasing number of potential H-bonding paths. With ring pruning, large ring populations are dramatically reduced and distributions can be identified to provide potential insight into the short- and intermediate-range structure in aqueous environments. Following the illustrated ring pruning examples shown in Figure 1, the common dihedral, angle, edge, and vertex criteria (CD, CA, CE, and CV, respectively) show an increasingly strong elimination of large rings in the H-bond network. Figure 4 shows the directional and normal ring count distributions in liquid water using TIP4P/Ice model at 298.15 K after applying each ring pruning option. Note that the ring counts are normalized by the maximum number of H-bonds in the simulation unit cell, so the contributing populations of the ring count distributions do not sum to 1. The CV and CE pruning options aggressively eliminate 6-membered or larger rings, resulting in overly simplified distributions that consist primarily of squares and pentagons. The highlighted CA method is most analogous to the approach used by Speedy et al.,^71^ and it produces a generally informative distribution that includes notable 6- and 7-membered ring information. The CD method further doubles the 6-membered ring population and enriches the 7- and 8-membered ring populations.

**Figure 4:**
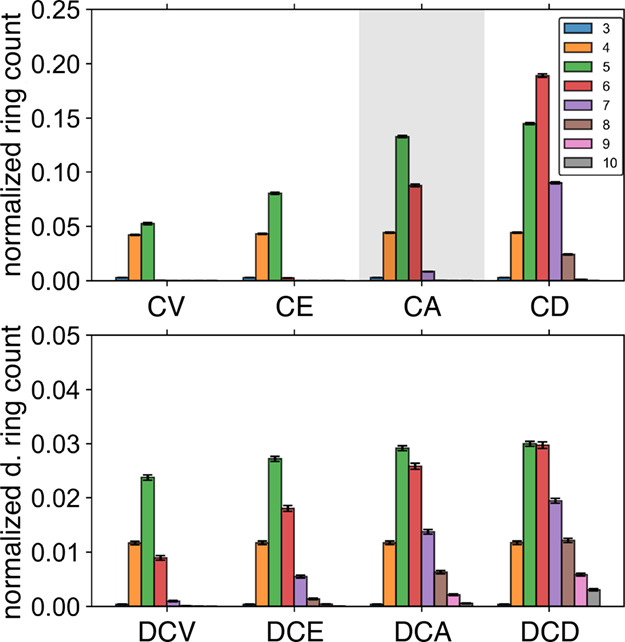
Ring pruning criteria are more aggressive with normal ring distributions than with directional ring distributions. Normal (upper panel) and directional (lower panel) ring distributions are shown for the different ring pruning criteria on a 1000 molecule TIP4P/Ice simulation at 298.15 K. Note the differing vertical scales for the normal and directional ring sets, with the directional ring counts proportionally smaller following *P*_D_.

Directional ring pruning methods eliminate rings more selectively than the normal ring pruning methods. This is because *P*_D_ naturally makes the directional rings an increasingly smaller subset of the overall ring population; note that the vertical scale in the lower panel is zoomed five times that of the upper panel to clearly show the distribution of populations in Figure 4. The lesser population of directional rings means that the rings are less likely to have common overlaps with a neighboring directional ring. This leads to a reduced elimination of rings overall. So while there are hardly any 8-membered rings to speak of in the CA pruned distribution, there is a moderately visible population of larger 10-membered rings in the DCA pruned distribution.

The chosen ring distribution in a water system should change as a function of the specific thermodynamic state. To investigate this, we monitored the broader DCA ring distribution as a function of temperature using the TIP4P/2005 and TIP4P/Ice water models, shown in Figure 5. In both temperature series, there is an enhanced ordering with decreasing temperature, enhanced primarily in the 5-, 6-, and 7-membered ring populations. This is to be expected, as decreasing thermal energy results in more stable and longer-lived H-bonds. While not shown, the analogous narrower CA ring distributions as a function of temperature are presented in the associated Supporting Information. The CA ring distributions show the same increase in ordering with decreasing temperature, though the increasing population is of primarily pentagons and hexagons at the expense of all other ring sizes.

**Figure 5:**
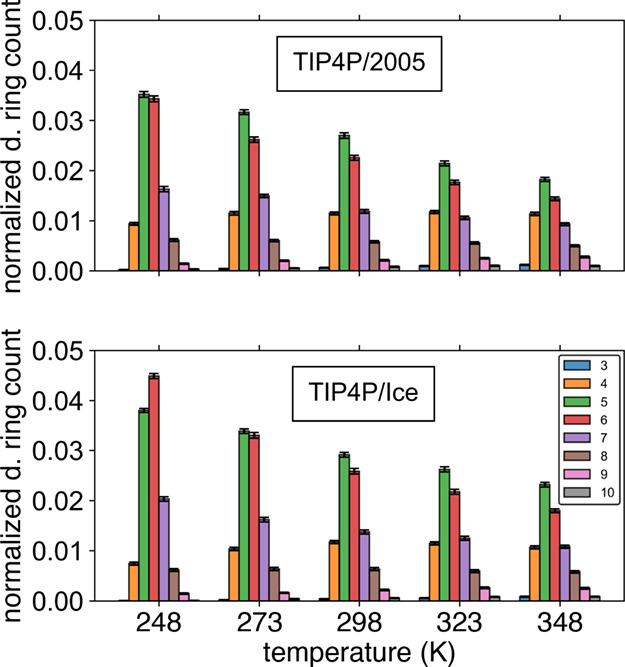
Directional ring distributions are taller and narrower with TIP4P/Ice than with TIP4P/2005 in simulations of water at the same temperatures. This indicates an offset of 25 K, in ordering behavior, similar to the difference in *T*_m_ values of 252 K for TIP4P/2005 versus 272 K for TIP4P/Ice. Ring distributions using the DCA pruning criteria are shown for simulations of the TIP4P/2005 (upper panel) and TIP4P/Ice (lower panel) water models as a function of temperature.

The relative populations of 5- and 6-membered rings in these DCA ring distributions serve as a visual proxy for system ordering. In addition to their increasing magnitude with decreasing temperature, there is an inversion in the relative populations of these rings near the melting points of the respective water models. The distribution profiles of TIP4P/2005 and TIP4P/Ice are quite similar, just with an offset where TIP4P/2005 needs to be 25 K colder than TIP4P/Ice to have an equivalent DCA ring distribution. This indicates that the liquid state ordering behavior of both water models is nearly equivalent after considering the 25 K temperature offset. Regardless of specific temperature or ring distribution analyses, the dominant ring populations are of 5- and 6-membered rings. The persistent strong population of 5-membered pentagons at and below the melting point is a sign of frustration in crystallization and is a signature of water’s ability to supercool and remain in a glassy state.^98^

Given the similarity in ring distributions for TIP4P/2005 and TIP4P/Ice and their apparent correspondence at melting temperatures, we decided to investigate the ring distributions for a series of common 3-site and 4-site water models at their melting temperatures. Figure 6 shows the CA and DCA ring distributions at the expected melting points (labeled) of several common 4-site water models.^87,99–101^ The ring distributions for 3-site water models are reported in the associated Supporting Information. The key point to note is the high similarity in the relative ring population across all these models at this state point. The DCA distributions all exhibit nearly equivalent 5- and 6-membered ring populations, making this analysis fairly convenient for characterizing the melting point of classical 4-site water models.

**Figure 6:**
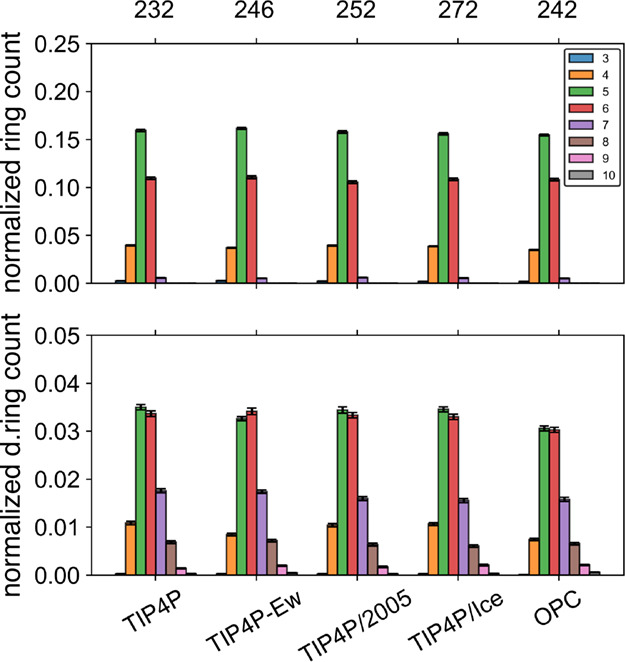
Water models show similar ring distributions at their melting temperatures. Normal (upper panel) and directional (lower panel) ring distributions using the CA ring pruning criteria from liquid simulations are shown for the TIP4P, TIP4P-Ew, TIP4P/2005, TIP4P/Ice, and OPC water models from liquid simulations performed at their respective melting temperatures.^87,99–101^

To quantitatively assess the similarity of these distributions and relative ordering among 4-site water models, as well as the TIP3P and SPC/E 3-site models, Table 2 reports the tetrahedral order parameter and RSF values at their corresponding *T*_m_ values.^100^ The 3-site water models exhibit slightly enhanced structural ordering over the 4-site water models, both in tetrahedrality and RSF. The 4-site models are very similar to one another, with both tetrahedrality and RSF values often overlapping within error. The consistent trends confirm that both metrics provide reliable and simplified quantitative assessment of ordering in liquid water.

**Table 2:** Tetrahedrality (*q*) and RSF Calculated for Liquid Simulations of Common Water Models at Their Respective Melting Temperatures

water model	*T*_m_ (K)	⟨*q*⟩	RSF
TIP3P	146	0.781(1)	0.367(1)
SPC/E	216	0.767(1)	0.347(2)
TIP4P	232	0.753(1)	0.316(2)
TIP4P-Ew	246	0.756(1)	0.316(2)
TIP4P/2005	252	0.745(1)	0.310(2)
TIP4P/Ice	272	0.745(1)	0.310(2)
OPC	242	0.744(1)	0.305(2)

### RSF Provides a Simple Quantitative Assessment of Order in Crystalline Systems

Ring enumeration algorithms require the systematic categorization of a complete network of H-bonds. It requires only little additional cost to calculate the tetrahedral order parameter for water, as defined by Errington and Debenedetti, alongside. In this way, we extract two simple metrics for assessing order in aqueous simulations. The primary differences between these metrics amount to the range of the response in liquid and supercooled water. Figure 7 shows the temperature dependence of both tetrahedrality and RSF, using 2 different ring pruning criteria, from the TIP4P/Ice simulations considered in Figure 5. Both tetrahedrality and RSF show a consistent decrease in ordering with increasing temperature in supercooled and liquid water, though the specific values calculated are different. Tetrahedrality hovers around 0.7 in the liquid state, whereas RSF is typically below 0.3. The long-range ordering of water in crystalline forms results in both tetrahedrality and RSF (regardless of the pruning method) approaching 1.0. The CV pruning method is shown in Figure 7 to highlight how RSF changes with the choice of the ring pruning method. Although the temperature-dependent behavior is similar, the CV approach is extremely aggressive and will significantly dampen the signal. The CA approach is generally more useful in building an ordering metric, and the signal behavior parallels tetrahedrality while better distinguishing liquid and supercooled forms of water from solid forms. While not shown, the CE pruning method falls between the CV and CA methods, as expected from the distributions shown in Figure 4. Using the CD pruning method will result in larger RSF values than the CA method, though this calculation will likely require enumerating rings larger than 10 members, making it more computationally prohibitive. Larger rings become more significant in the ring distribution when using CD pruning, again as shown in Figure 4.

**Figure 7:**
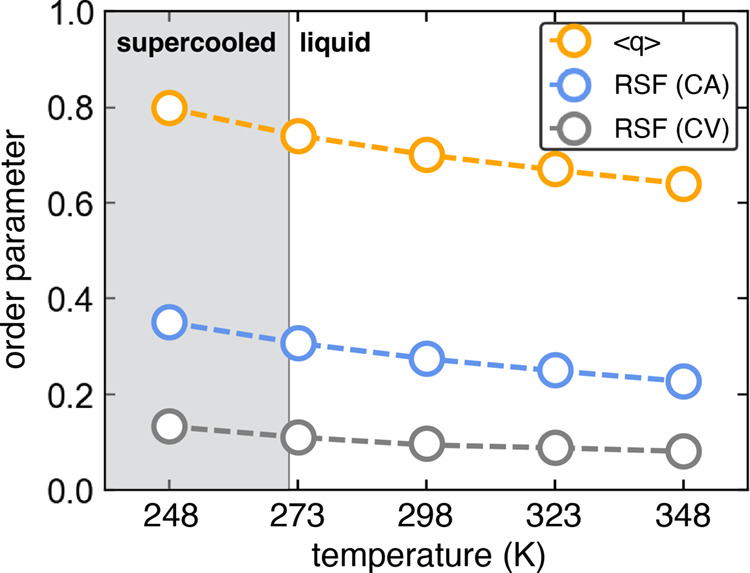
Tetrahedral order parameter and RSF metric derived from CA and CV pruning show a similar decreasing trend as a function of increasing temperature in supercooled and liquid water using the TIP4P/Ice water model. While both tetrahedrality and RSF approach 1.0 in crystalline water, the values from RSF are notably smaller, indicating RSF has an increased range of response between the crystalline and liquid states.

While ice Ih or ice Ic are expected to have highly tetrahedral structures with tetrahedrality and RSF both near 1.0, other ice polymorphs can be more complex. Consider ice VI and XII as shown in Table 3. With tetrahedrality values below 0.7 and 0.8 respectively, less dense supercooled water can have greater calculated tetrahedrality than the water in these structures; the 248 K temperature supercooled water in Figure 7 has a value greater than 0.8. The lowered tetrahedral order parameter in these and other dense ice polymorphs is due to distortion of the ideal tetrahedral H-bonding arrangements when the crystals are formed under extreme pressures. Such intrinsic distortions do impact the extended H-bonding network, as RSF also sees a notable decline from 1.0 in many of these same polymorphs. With ice III, RSF is lower than supercooled liquid values at low temperatures, the same behavior is seen with the tetrahedral order parameter with other high-density ice polymorphs.

**Table 3:** Average Tetrahedrality, ⟨*q*⟩, and RSF Using CA Pruning in Proton Ordered and Proton Disordered Ice Polymorphs

ice phase	⟨*q*⟩	RSF
Ih	0.9979(10)	1.000
Ic	0.9982(8)	1.000
XI_af_	0.9977(2)	1.000
VII	0.36(6)	1.000
VIII	0.33(1)	1.000
III	0.86(4)	0.333
IX	0.86(5)	0.333
VI	0.69(3)	0.600
XV	0.69(3)	0.600
XII	0.79(10)	0.667
XIV	0.78(10)	0.667

Additionally, Table 3 anomalously shows that the proton ordered/disordered ice VII/VIII pair exhibits low water ordering values, despite the nearly perfect tetrahedrality in ice Ic. The interlocking of two noninteracting ice Ic structures creates the highly dense body-centered cubic ice VII, in which every water is in contact with eight neighboring water molecules. The calculation of tetrahedrality only involves a loop over four nearest neighbors about each particle, so eight equidistant water molecules are not well handled without knowledge of the H-bonding of the central water and its neighbors. Prioritizing the selection of four neighbors can be done after first sorting by the standard geometric H-bond definition,^78^ and this leads to respective proton ordered ice VII and proton disordered ice VIII tetrahedrality values of 0.99(6) and 0.999(1). While calculations of tetrahedral order in ice VII/VIII are not overly common, incorporating knowledge of H-bonding beyond simple tetrahedral ordering is likely necessary for well-behaved results. RSF yields an ideal ordered value of 1.0 for both crystal forms, where every water molecule forms, on average, two unique rings.

The RSF values in Table 3 show the same decrease in order observed with tetrahedrality for select high-pressure ice polymorphs. The reported RSF value is built from the calculated ring distribution, which includes additional information about the types of rings that contribute to the summation. Figure 8 presents the CA pruned ring distributions for three forms of high-pressure ice alongside supercooled water. While ice III has the lowest RSF value of the four states of water, it is actually quite ordered in that only 5-membered rings exist after pruning rings that share a common angle. The supercooled liquid at 248 K shows a higher RSF value than ice III, but the presence of a smoother distribution spanning multiple neighboring ring sizes indicates a lack of long-range order, characteristic of a less ordered material. Ice VI and ice XII have similar RSF values, though ice VI is a proportional set of 4- and 8-membered rings, while ice XII only contains heptagons after pruning. In general, RSF is a simplified presentation of these distributions, one that loses information intrinsic to the full distribution used in the summation.

**Figure 8:**
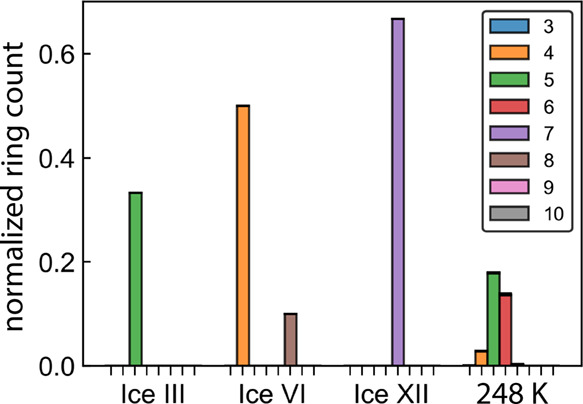
Ring distributions using CA pruning for high pressure ice III, ice VI, and ice XII are more selective for specific ring types than supercooled water at 248 K. In ice structures, distributions typically contain only one or two ring types, whereas in a liquid or supercooled state, there is a greater variety.

### Ordering in Ice Ih and Its Proton Ordered Polymorphs

Normal ice Ih is proton disordered, yet this does not stop the crystal from having near-ideal tetrahedral arrangements of water molecules.^13,102^ Ice Ih has two proton ordered counterpart structures in ferroelectric (*Cmc*2_1_ symmetry) ice XI and antiferroelectric (*Pna*2_1_ symmetry) ice XI. Antiferroelectric ice has eight water molecules per unit cell, and it possesses a zero magnitude dipole moment upon summation of the individual water moment vectors in the unit cell shown in Figure 9a.^103–105^ Ferroelectric ice XI instead has four water molecules per unit cell (two unit cells are pictured) and exhibits a strong electrostatic force due to extended alignment of water dipoles along the vertical axis of the unit cells. These unit cells tile to form crystals with percolating loops in all 6-membered rings. The formation of a percolating loop of H-bonds involves one or more water molecules that donate two H-bonds in the ring (the water with both orange and blue H atoms in Figure 9b) and one or more water molecules that accept two H-bonds in the ring.^106^ Given this distinct behavior, ring directionality can accordingly play a role in identifying and characterizing the macroscopic polarization and dielectric properties of proton ordered ice phases.

**Figure 9:**
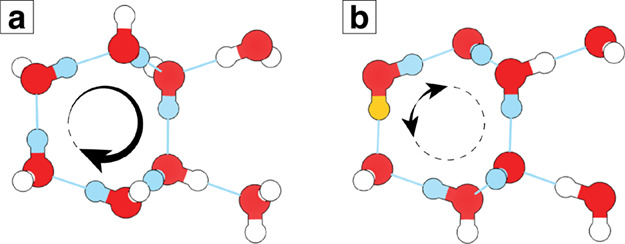
Smallest repeating units in proton ordered ice XI form hexagonal rings. (a) A directional ring is formed in antiferroelectric ice XI, and (b) percolating loops in ferroelectric ice XI arise due to the presence of water molecules acting only as a H-bond donor or acceptor within the ring.

To explore how directional rings can be used to identify proton ordering behavior, we analyzed both proton ordered and proton disordered ice Ih simulation cells with the same number of water molecules, 1024 (Figure 10a,d). Without considering directionality of the H-bonding, each of these crystals will have 2048 6-membered rings, a value twice the number of water molecules. While larger rings, like those with 8 and 10 water molecules, are present in the crystal, the CA pruning method eliminates all rings larger than these 6-membered rings (Figure 10b,e). Directional rings, however, occur randomly in proton disordered ice crystals, as seen in Figure 10c. While most of the 8-member and larger rings are eliminated by CA pruning, there is the possibility for some larger directional rings to survive, one identified with the light green dot in Figure 10c and Table 4. Note that the tabulated values come from averaging over 15 unique proton disordered crystals generated from the GenIce2 tool.^94^ With some proton ordered ices, the occurrence of directional rings will be uniformly present, as in the 512 observed in this antiferroelectric ice XI simulation cell. These rings are uniformly absent, with 0 in a ferroelectric ice XI simulation cell due to percolating loops as shown in Figure 9.

**Figure 10:**
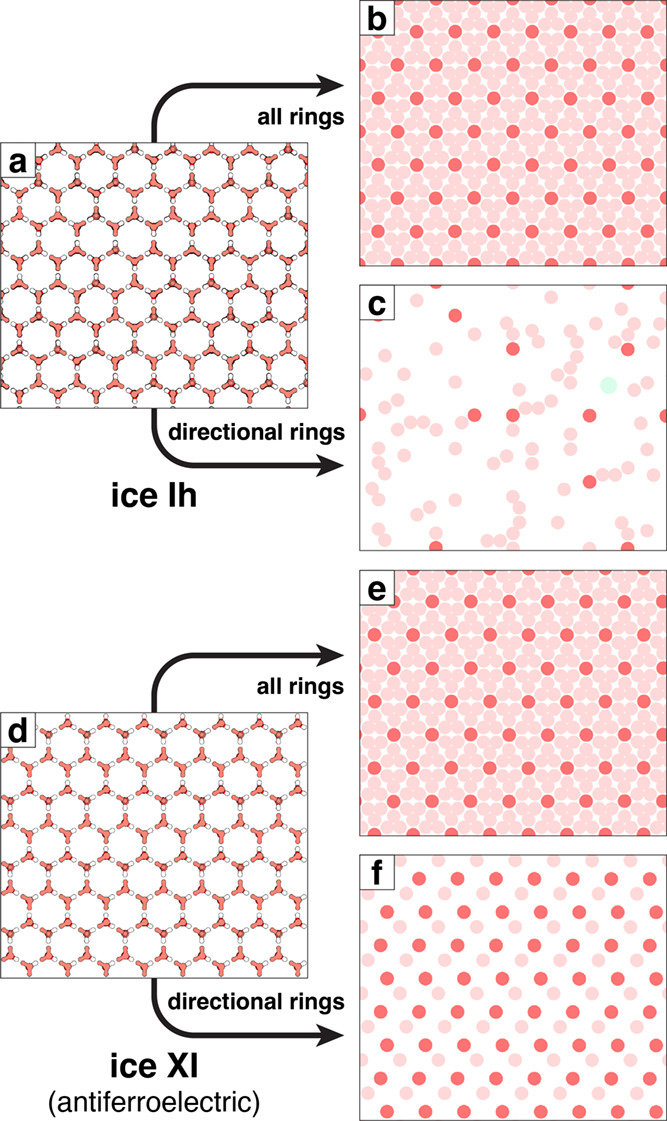
Visualizing the basal plane of ice Ih and ice XI highlights how directional rings can help identify proton ordering in ice. (a) A ball-and-stick representation of a proton disordered ice Ih crystal, (b) a representation with red circles located at the center of all hexagonal rings in a 1 Å thick slab of the crystal, with darker red circles in the top layer of the slab, and (c) the same as (b) but only showing directional rings. Panels (d), (e), and (f) are the same as (a), (b), and (c), but for antiferroelectric ice XI with the most apparent differences being the (c) and (f) directional ring panels, where proton ordering is apparent in (f).

**Table 4:** Directional Ring Counts in Simulation Cells Containing 1024 Water Molecules in Proton Disordered Ice Ih, Antiferroelectric (af) Ice XI, and Ferroelectric (f) Ice XI

ice polymorph	⟨6⟩	⟨8⟩
Ih	361(3)	12(1)
XI_af_	512	0
XI_f_	0	0

### Ordering in Ice Ic and Ice VII/VIII

Proton ordering is important for more than just hexagonal ice. Other distinguishable ice polymorphs, such as the very dense ice VII and ice VIII, exhibit clear order–disorder transitions. The ice VII (and VIII) crystal is built from two interpenetrating ice Ic crystals. The increased density of this packing is visible by the difference in slab surface rings depicted with the denser array of red circles in Figure 11c (ice VII) over that seen in Figure 11a (ice Ic). Both ice Ic and ice VII are proton disordered crystals, and we see a similar random distribution of directional rings present (Figure 11b,d) as observed in ice Ih. The population of directional rings is actually observed to be proportionally identical (Table 5) across a set of 15 random crystals from GenIce2, proportional with respect to the number of water molecules in the simulation cells. We do not picture ice VIII in Figure 11 because, similar to ferroelectric ice XI, no directional rings are present in the crystal (see Table 5).

**Figure 11:**
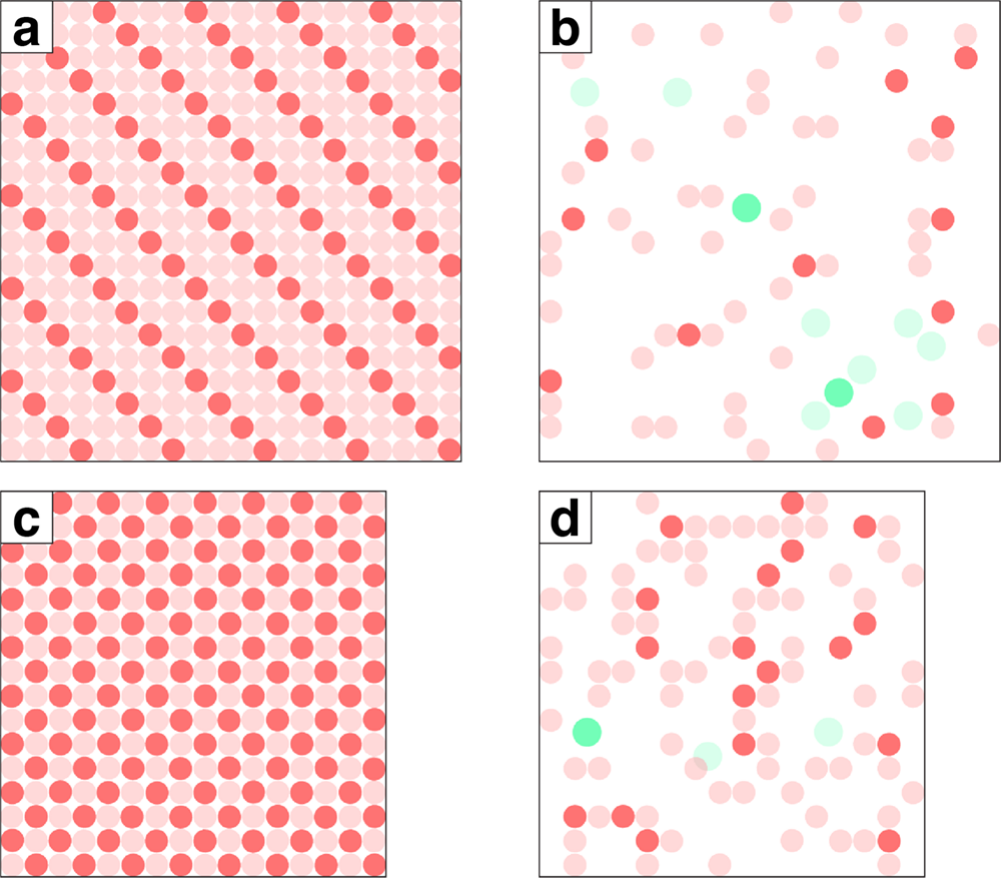
Rings present in a 1 Å thick slab of (a) ice Ic shows it to be significantly less dense than (c) ice VII via the less dense array of red circles. When only considering directional rings in (b) ice Ic and (d) ice VII, the resulting distributions are random and occasionally contain octagons in addition to the typical hexagons.

**Table 5:** Directional Ring Counts in Proton Disordered Ice Ic (1000 Water Molecule Simulation Cell) Crystals and Highly Dense Ice VII and Ice VIII (1024 Molecule) Crystals

ice polymorph	⟨6⟩	⟨8⟩
Ic	350(3)	16(1)
VII	361(2)	16(1)
VIII	0	0

### Tracking Water Structural Ordering during Crystallization

One of the clearest order–disorder transitions can be seen in the transition between crystalline and liquid/supercooled phases. Tetrahedrality is quite useful for monitoring such phase-transition processes, and RSF from ring distributions should provide similar insights. To investigate this, we performed a series of phase-coexistence simulations to study the crystal-seed induced crystallization of supercooled water (260 K and 1 atm) using the TIP4P/Ice water model. Figure 12 shows a short equilibrated biphasic system, with water molecules color-coded by their individual tetrahedrality values. The crystal phase exhibits a high degree of tetrahedrality and appears deep blue, contrasting with the less ordered liquid water phase, which ranges from lighter blue to white. Occasionally, a few water molecules may be highly unstructured, like an ideal gas, and these molecules are colored red on this color scale. Averaging the individual molecule tetrahedrality values gives an overall tetrahedrality, ⟨*q*⟩. We used both ⟨*q*⟩ and RSF to monitor the crystal growth on the secondary prismatic plane of the seed crystal, the plane of the crystal exposed to the supercooled water.

**Figure 12:**
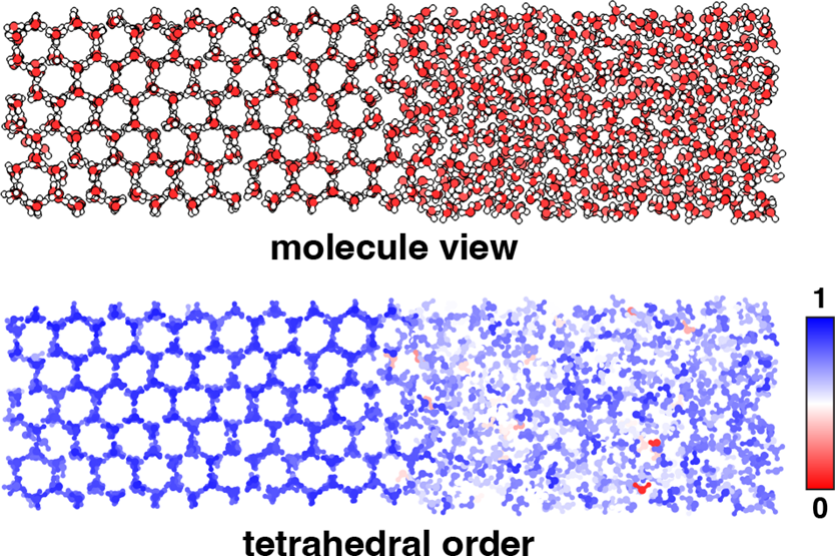
Tetrahedral order parameter highlights the difference in local structural ordering between ice and liquid water, as observed in a snapshot of a biphasic system using the TIP4P/Ice water model at 260 K. The upper panel shows a molecule view and in the lower panel, the water molecules are colored based on the tetrahedrality. Dark blue coloring represents highly structured water with a tetrahedrality value near 1, and red indicates unstructured water with a value close to 0.

The three panels in Figure 13 show three different simulation states, all under the same conditions. In purely supercooled simulations on the left, RSF reports a significantly lower average value (around 0.3) than tetrahedrality (around 0.8). In crystalline simulations at the same temperature on the right, the situation is flipped with RSF reporting a value near 1.0 while tetrahedrality’s average value hovers near 0.95. In seeded simulations in the middle panel, the two comparable ordering metrics exhibit a systematic progression from a lower-order supercooled state to a higher-order crystalline state during crystallization, and the crystal growth rate can be calculated over this slope. Interestingly, the RSF does not appear to fully plateau near 40 ns, as seen with tetrahedrality. RSF shows a logarithmic-like approach to an average value of 1.0 between 40 and 70 ns. This is a period where the crystal appears complete, but defects in the crystal are undergoing an annealing process. Rings in the H-bond network are more sensitive to defects than tetrahedrality because loss of H-bonds will readily eliminate rings in RSF, but this will only appear as a potential moderate skew in the tetrahedrality. This indicates that RSF more clearly resolves the subtleties of this phase transition due to its wider range of values. RSF is a useful metric for monitoring order during nucleation and crystallization in supercooled water, offering potential advantages over the commonly used tetrahedrality metric.

**Figure 13:**
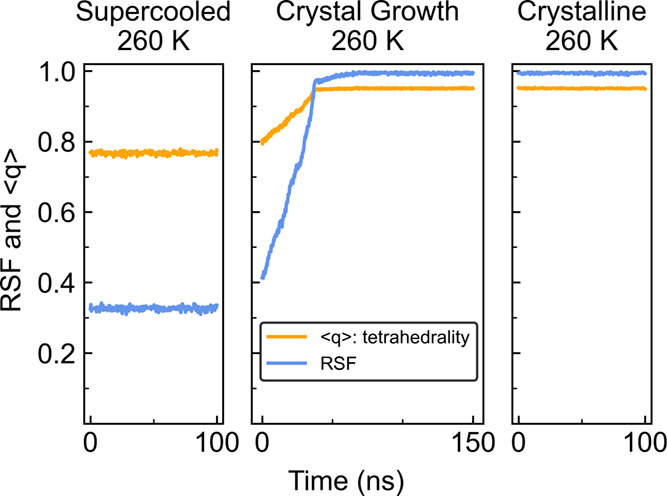
RSF, like tetrahedrality, works as an order parameter for monitoring the progress of crystal growth. The trend of water ordering in supercooled liquid (left), crystal growth (middle), and complete crystallization (right) highlights the wider response range with RSF using CA pruning, and one can observe defect annealing with RSF between 40 and 70 ns in this particular growth.

## Conclusion

The H-bond network in condensed phase simulated water reveals the state, behavior, and order of the system. In this study, we explored closed-path formation of H-bond sequences in ring distributions, both with and without considering the H-bond directionality. While systematically identifying and pruning H-bond ring paths across liquid, supercooled, and crystalline water systems, we devised a ring summation factor (RSF) metric, which is a simplified measure for the quantification of order, similar to the average tetrahedral order parameter.

The ring distribution patterns, RSF, and tetrahedrality all provide unique insights into simulations involving water. For example, there is a clear consistency in the observed ring distributions at the melting temperature of four-point water models, such that simply monitoring the population shift of directional rings between majority 5- and 6-membered rings can identify the transition point. RSF characterizes liquid and supercooled states under various conditions, similar to what is possible with tetrahedrality. Additionally, the RSF metric enables clear distinction of order–disorder transitions in water simulations alongside visualization of the kinetics and subprocesses involved in crystal growth over phase transitions. Finally, we can distinguish proton ordered from proton disordered ice polymorphs by enumerating directional rings, visible in POV-Ray fingerprint images of directional ring size and location. By considering H-bond network topology, it is possible to more clearly characterize the behavior of water models, probe their distinguishing anomalous properties, and potentially more clearly identify the driving forces present in more structured liquid water in comparison to normal liquids.

## Supplementary Material



## Data Availability

The *nucleation_tracker* python code for calculation of ring distributions and tetrahedrality is freely available for download from a publicly accessible GitHub repository under the “nucleation_tracker” project name, presently at the github.com/FennellLab/nucleation_tracker web address.

## Notes

Data present in the figures of the main article and additional information supporting the findings of this study (PDF)
