# Supplementary material for: Assessing Order in Liquid, Supercooled, and Crystalline Water
Source: J Phys Chem B. 2026 May 2;130(19):5037–49. doi: 10.1021/acs.jpcb.5c08791 (PMC13181771; doi:10.1021/acs.jpcb.5c08791)
Supplement: Supplementary file 1 [file jp5c08791_si_001.pdf]

Supplementary Information: Assessing Order in Liquid, Supercooled, and Crystalline Water

Rajendra Maharjan[1,2*], Casey Williamson[1], and Christopher J. Fennell[1*]

[1]Department of Chemistry, Oklahoma State University,
Stillwater, Oklahoma, 74078, USA
[2]Department of Chemistry, Grinnell College, Grinnell, IA, 50112, USA
*To whom correspondence should be addressed; E-mail: maharjanra@grinnell.edu or christopher.fennell@okstate.edu

April 26, 2026

The supplementary material provides additional data and analyses that support the findings presented in the main article. This includes detail results obtained using various water models, as well as comprehensive comparisons of directional and non-directional ring distributions. These supplementary figures and tables illustrate further insight into the structural characteristics observed, ensuring the transparency and reproducibility of our computational approach.

List of Figures and Tables

Table S1: **Enumeration of directional rings using TIP4P/2005 water model at 298.15 K**

**Pruning criteria**	⟨3⟩	⟨4⟩	⟨5⟩	⟨6⟩	⟨7⟩	⟨8⟩	⟨9⟩	⟨10⟩
D.C.V.	0.0006	0.0114	0.0223	0.0082	0.0012	0.0001	0	0
D.C.E.	0.0006	0.0114	0.0255	0.0162	0.005	0.0016	0.0003	0
D.C.A.	0.0006	0.0115	0.0270	0.0225	0.0118	0.0058	0.0021	0.0008
D.C.D.	0.0006	0.0115	0.0276	0.0259	0.0170	0.0104	0.0061	0.0031

The average standard error is within 0.0003.

Table S2: **Enumeration of non-directional rings using TIP4P/2005 water model at 298.15 K**

**Pruning criteria**	⟨3⟩	⟨4⟩	⟨5⟩	⟨6⟩	⟨7⟩	⟨8⟩	⟨9⟩	⟨10⟩
C.V.	0.0039	0.0416	0.0429	0.0004	0	0	0	0
C.E.	0.0039	0.0432	0.0675	0.0031	0	0	0	0
C.A.	0.0039	0.0449	0.1174	0.0764	0.0096	0.0003	0	0
C.D.	0.0039	0.0450	0.1294	0.1611	0.0837	0.0253	0.0023	0

The average standard error is within 0.0004.

Table S3: **Enumeration of directional rings using TIP4P/Ice water model at 298.15 K**

**Pruning criteria**	⟨3⟩	⟨4⟩	⟨5⟩	⟨6⟩	⟨7⟩	⟨8⟩	⟨9⟩	⟨10⟩
D.C.V.	0.0004	0.0116	0.0238	0.0089	0.0010	0.0001	0	0
D.C.E.	0.0004	0.0117	0.0272	0.0180	0.0055	0.0014	0.0004	0
D.C.A.	0.0004	0.0117	0.0291	0.0258	0.0137	0.0063	0.0021	0.0006
D.C.D.	0.0004	0.0117	0.0299	0.0297	0.0194	0.0121	0.0058	0.0031

The average standard error is within 0.0003.

Table S4: **Enumeration of non-directional rings using TIP4P/Ice water model at 298.15 K**

Pruning criteria	⟨3⟩	⟨4⟩	⟨5⟩	⟨6⟩	⟨7⟩	⟨8⟩	⟨9⟩	⟨10⟩
C.V.	0.0028	0.0421	0.0524	0.0002	0	0	0	0
C.E.	0.0028	0.0430	0.0804	0.0025	0	0	0	0
C.A.	0.0028	0.0442	0.1329	0.0875	0.0084	0.0001	0	0
C.D.	0.0028	0.0442	0.1448	0.1890	0.0901	0.0240	0.0011	0

The average standard error is within 0.0004.

Table S5: **Directional ring distribution using CA pruning method at different temperatures using TIP4P/2005 water model**

Temperature (K)	⟨3⟩	⟨4⟩	⟨5⟩	⟨6⟩	⟨7⟩	⟨8⟩	⟨9⟩	⟨10⟩
248.15	0.0002	0.0094	0.0352	0.0343	0.0163	0.0061	0.0014	0.0003
273.15	0.0004	0.0115	0.0317	0.0262	0.0149	0.0060	0.0020	0.0005
298.15	0.0006	0.0115	0.0270	0.0225	0.0118	0.0058	0.0021	0.0008
323.15	0.0010	0.0117	0.0214	0.0176	0.0106	0.0056	0.0025	0.0010
348.15	0.0012	0.0114	0.0182	0.0144	0.0093	0.0050	0.0027	0.0010

The average standard error is within 0.0003.

Table S6: **Non-directional ring distribution using CA pruning method at different temperatures using TIP4P/2005 water model**

Temperature (K)	⟨3⟩	⟨4⟩	⟨5⟩	⟨6⟩	⟨7⟩	⟨8⟩	⟨9⟩	⟨10⟩
248.15	0.0021	0.0375	0.1594	0.1124	0.0054	0	0	0
273.15	0.0030	0.0429	0.1387	0.0889	0.0071	0.0001	0	0
298.15	0.0039	0.0449	0.1174	0.0764	0.0096	0.0003	0	0
323.15	0.0046	0.0443	0.0972	0.0650	0.0117	0.0006	0	0
348.15	0.0057	0.0421	0.0818	0.0551	0.0132	0.0013	0.0001	0

The average standard error is within 0.0004.

Table S7: **Directional ring distribution using CA pruning method at different temperatures using TIP4P/Ice water model**

**Temperature (K)**	$\langle 3 \rangle$	$\langle 4 \rangle$	$\langle 5 \rangle$	$\langle 6 \rangle$	$\langle 7 \rangle$	$\langle 8 \rangle$	$\langle 9 \rangle$	$\langle 10 \rangle$
248.15	0	0.0074	0.0380	0.0449	0.0203	0.0061	0.0014	0.0001
273.15	0.0002	0.0103	0.0338	0.0330	0.0162	0.0063	0.0016	0.0004
298.15	0.0004	0.0117	0.0291	0.0258	0.0137	0.0063	0.0021	0.0006
323.15	0.0006	0.0114	0.0262	0.0218	0.0124	0.0059	0.0026	0.0008
348.15	0.0008	0.0106	0.0232	0.0179	0.0108	0.0058	0.0025	0.0008

The average standard error is within 0.0003.

Table S8: **Non-directional ring distribution using CA pruning method at different temperatures using TIP4P/Ice water model**

**Temperature (K)**	$\langle 3 \rangle$	$\langle 4 \rangle$	$\langle 5 \rangle$	$\langle 6 \rangle$	$\langle 7 \rangle$	$\langle 8 \rangle$	$\langle 9 \rangle$	$\langle 10 \rangle$
248.15	0.0012	0.0286	0.1792	0.1378	0.0036	0	0	0
273.15	0.0020	0.0385	0.1569	0.1045	0.0059	0.0001	0	0
298.15	0.0028	0.0442	0.1329	0.0875	0.0084	0.0001	0	0
323.15	0.0036	0.0436	0.1168	0.0762	0.0100	0.0004	0	0
348.15	0.0046	0.0431	0.1018	0.0661	0.0116	0.0006	0	0

The average standard error is within 0.0004.

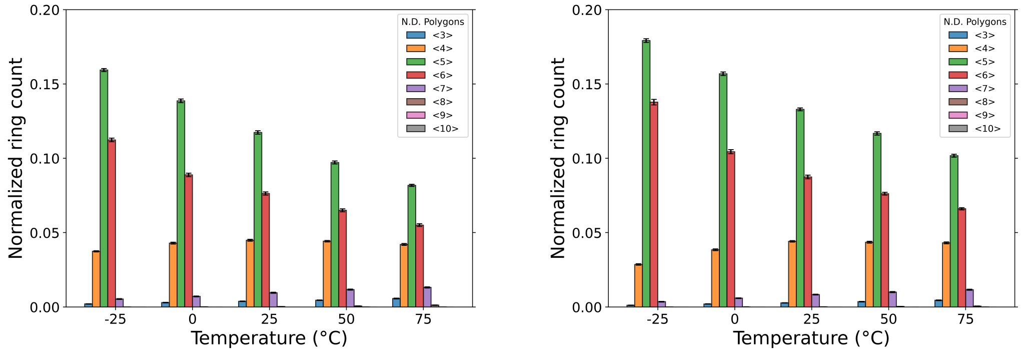

(a) (b)

Figure S1: The non-directional ring distributions are taller with TIP4P/Ice than with TIP4P/2005 in simulations of water at the same temperatures. Non-directional ring distributions using the CA pruning criteria are shown for simulations of the (a) TIP4P/2005 *left* and (b) TIP4P/Ice *right* water models as a function of temperature.

We observed that 3-site and 4-site water models each exhibit similar ring distributions and hence similar network topologies in both liquid and supercooled water at their respective melting points and at temperatures scaled relative to their melting temperatures.

Table S9: **Directional ring distribution using CA pruning criteria at the respective melting points of different water models**

**Water models**	⟨3⟩	⟨4⟩	⟨5⟩	⟨6⟩	⟨7⟩	⟨8⟩	⟨9⟩	⟨10⟩
SPC/E	0	0.0053	0.0287	0.0364	0.0196	0.0073	0.0020	0.0003
TIP3P	0	0.0020	0.0252	0.0346	0.0195	0.0087	0.0041	0.0002
TIP4P	0.0002	0.0108	0.0350	0.0336	0.0176	0.0068	0.0014	0.0002
TIP4P/Ew	0.0002	0.0084	0.0326	0.0341	0.0174	0.0072	0.0019	0.0004
TIP4P/2005	0.0002	0.0104	0.0344	0.0333	0.0159	0.0063	0.0017	0.0002
TIP4P/Ice	0.0002	0.0106	0.0345	0.0330	0.0155	0.0060	0.0021	0.0003
OPC	0.0001	0.0074	0.0306	0.0303	0.0158	0.0065	0.0021	0.0006

The average standard error is within 0.0003.

Table S10: **Non-directional ring distribution using CA pruning method at the respective melting points of different water models**

**Water models**	$\langle 3 \rangle$	$\langle 4 \rangle$	$\langle 5 \rangle$	$\langle 6 \rangle$	$\langle 7 \rangle$	$\langle 8 \rangle$	$\langle 9 \rangle$	$\langle 10 \rangle$
SPC/E	0.0022	0.0278	0.1760	0.1375	0.0041	0	0	0
TIP3P	0.0014	0.0207	0.1834	0.1586	0.0032	0	0	0
TIP4P	0.0025	0.0394	0.1593	0.1095	0.0056	0	0	0
TIP4P/Ew	0.0025	0.0370	0.1614	0.1104	0.0051	0.0001	0	0
TIP4P/2005	0.0022	0.0392	0.1578	0.1053	0.0059	0	0	0
TIP4P/Ice	0.0020	0.0384	0.1558	0.1083	0.0054	0	0	0
OPC	0.0020	0.0348	0.1547	0.1080	0.0051	0	0	0

The average standard error is within 0.0004.

Table S11: **Trend of tetrahedral order parameter, q, in liquid and supercooled water as a function of temperature using TIP4P/2005 and TIP4P/Ice water models**

**Temperature (K)**	**TIP4P/Ice, $\langle q \rangle$**	**TIP4P/2005, $\langle q \rangle$**
248.15	0.798(1)	0.7525(1)
273.15	0.742(1)	0.7086(1)
298.15	0.701(1)	0.6722(1)
323.15	0.671(1)	0.6385(1)
348.15	0.641(1)	0.6104(1)

Table S12: **Trend of pentagons and hexagons in liquid and supercooled water as a function of temperature using TIP4P/2005 and TIP4P/Ice water models**

**Temperature (K)**	**TIP4P/Ice**		**TIP4P/2005**	
	$\langle \mathbf{5} \rangle$	$\langle \mathbf{6} \rangle$	$\langle \mathbf{5} \rangle$	$\langle \mathbf{6} \rangle$
248.15	0.159(1)	0.112(1)	0.179(1)	0.138(1)
273.15	0.139(1)	0.089(1)	0.157(1)	0.104(1)
298.15	0.117(1)	0.076(1)	0.133(1)	0.087(1)
323.15	0.097(1)	0.065(1)	0.117(1)	0.076(1)
348.15	0.082(1)	0.055(1)	0.102(1)	0.066(1)

Table S13: **Tetrahedrality, q, and ring summation factor, RSF, in liquid and supercooled water using TIP4P/Ice water models**

**Temperature (K)**	**Tetrahedral order, ⟨q⟩**	**RSF**
248.15	0.798(1)	0.350(2)
273.15	0.742(1)	0.308(2)
298.15	0.701(1)	0.276(2)
323.15	0.671(1)	0.251(2)
348.15	0.641(1)	0.228(1)

Table S14: **Tetrahedrality, q, and ring summation factor, RSF, in liquid and supercooled water using TIP4P/2005 water models**

**Temperature (K)**	**Tetrahedral order, $\langle q \rangle$**	**RSF**
248.15	0.752(1)	0.317(2)
273.15	0.709(1)	0.281(2)
298.15	0.672(1)	0.252(2)
323.15	0.638(1)	0.223(2)
348.15	0.610(1)	0.199(1)

Table S15: **Enumeration of rings in ice III, ice VI, ice XII, and supercooled water at 248 K using TIP4P/Ice water model**

**Pruning criteria**	$\langle 3 \rangle$	$\langle 4 \rangle$	$\langle 5 \rangle$	$\langle 6 \rangle$	$\langle 7 \rangle$	$\langle 8 \rangle$	$\langle 9 \rangle$	$\langle 10 \rangle$
Ice III	0.	0	0.3333	0	0	0	0	0
Ice VI	0	0.5000	0	0	0	0.1000	0	0
Ice XII	0	0	0	0	0.6667	0	0	0
248 K	0.0012	0.0286	0.1792	0.1378	0.0036	0	0	0

The average standard error for supercooled water at 248 K is within 0.0005.
